# Constrained Pattern of Viral Evolution in Acute and Early HCV Infection Limits Viral Plasticity

**DOI:** 10.1371/journal.pone.0016797

**Published:** 2011-02-08

**Authors:** Katja Pfafferott, Silvana Gaudieri, Axel Ulsenheimer, Ian James, Malte Heeg, David Nolan, Mina John, Andri Rauch, Simon Mallal, Andrew Lucas, Paul Klenerman, Helmut M. Diepolder, Michaela Lucas

**Affiliations:** 1 Centre for Clinical Immunology and Biomedical Statistics, Institute for Immunology & Infectious Diseases, Murdoch University and Royal Perth Hospital, Perth, Australia; 2 School of Anatomy and Human Biology and Centre for Forensic Science, University of Western Australia, Perth, Australia; 3 Medical Department II and Institute for Immunology, Ludwig-Maximilians-University Munich, Munich, Germany; 4 University Hospital Berne and University of Berne, Berne, Switzerland; 5 Nuffield Department of Clinical Medicine, Oxford University, Oxford, United Kingdom; Institut Pasteur, France

## Abstract

Cellular immune responses during acute Hepatitis C virus (HCV) and HIV infection are a known correlate of infection outcome. Viral adaptation to these responses via mutation(s) within CD8+ T-cell epitopes allows these viruses to subvert host immune control. This study examined HCV evolution in 21 HCV genotype 1-infected subjects to characterise the level of viral adaptation during acute and early HCV infection. Of the total mutations observed 25% were within described CD8+ T-cell epitopes or at viral adaptation sites. Most mutations were maintained into the chronic phase of HCV infection (75%). The lack of reversion of adaptations and high proportion of silent substitutions suggests that HCV has structural and functional limitations that constrain evolution. These results were compared to the pattern of viral evolution observed in 98 subjects during a similar phase in HIV infection from a previous study. In contrast to HCV, evolution during acute HIV infection is marked by high levels of amino acid change relative to silent substitutions, including a higher proportion of adaptations, likely reflecting strong and continued CD8+ T-cell pressure combined with greater plasticity of the virus. Understanding viral escape dynamics for these two viruses is important for effective T cell vaccine design.

## Introduction

The initial cellular immune response to the Hepatitis C virus (HCV) plays an important role in viral control following infection [1], [2], [3], [4], [5], [6]. Studies in both humans and surrogate animal models indicate that a strong and durable CD8^+^ T-cell response aids in controlling infection [7], [8], however HCV mutations within immune targets (epitopes) lead to viral escape (adaptations) from the host's immune response [9], [10], [11], [12], [13]. Information on the dynamics of viral adaptation during the critical acute phase of HCV infection is still limited due to the lack of large acute HCV infection clinical cohorts and the small number of known HCV T cell epitopes.

Previous studies examining viral evolution during acute and early HCV infection on a small number of subjects showed that the proportion of mutations likely to be associated with CD8^+^ T-cell immune pressure varies from 11–25% [9], [13]. These results contrast with those from HIV-1 and SIV studies which suggest that the proportion of mutations associated with CD8^+^ T-cell immune pressure during acute infection can be greater than 50% and is a major driving force in HIV evolution [14]–[16]. Furthermore, a substantial number of early mutations in HIV are reversions of viral adaptations to non-adapted or wildtype virus in the absence of the selecting immune pressure [14] suggesting a fast rate of forward and reverse mutations relative to HCV. The pattern of viral adaptation in HIV and HCV are often treated as comparable in discussions of successful anti-viral host immune responses and vaccine design. However, a direct comparison between the patterns of viral evolution in these two viruses in the critical acute phase of infection has not been performed. This comparison is hampered by the discrepancy between the numbers of known T-cell epitopes for the two viruses. There is a much larger number of known T-cell epitopes ([17], Los Alamos http://hcv.lanl.gov [18] and IEDB [19] http://www.immuneepitope.org) that cover the HIV proteome at a greater density than within the HCV proteome. Accordingly, a direct comparison of the evolutionary dynamics of the two viruses must attempt to account for these differences.

The selection of viral epitopes presented to CD8^+^ T-cells is restricted by the repertoire of human leucocyte antigen (HLA) class I molecules present in the infected host. To date, the number of known T-cell epitopes for HCV is relatively limited and there is an over-representation of epitopes restricted to common HLA types (e.g. HLA-A2). More recently, as an alternative approach to conventional cellular analyses to identify HCV epitopes, population-based genetic studies have found statistically significant associations between polymorphisms within the viral genome and specific HLA types [20], [21], [22]. These putative viral adaptation sites are likely to mark *in-vivo* T-cell targets and indeed several of these sites have been shown to fall within known T-cell epitopes with the appropriate HLA-restriction and escape pattern [20], [21], [22]. These viral adaptation sites may represent additional sites of HCV-specific T-cell targets for a broader range of HLA types present in human populations that can complement the existing databases of published HCV-specific T-cell epitopes and provide a better framework to study HCV adaptation to HLA-restricted immune responses during infection.

In this study we examine the dynamics of viral evolution and specifically viral adaptation during acute and chronic HCV infection in sequential sequences from 21 HCV-genotype 1 infected individuals with different outcomes. We circumvent, to some extent, the issue of limited published HCV epitopes by including in the analysis putative viral adaptation sites from genetic-based studies [20], [22]. We then compare viral evolution during the critical acute to chronic infection phase between HIV and HCV utilising HIV sequences obtained from a published longitudinal study on acute HIV infection (n = 98; [14]) which also utilised viral adaptation sites from genetic-based studies. Though the HIV studies have had much larger cohort sizes, this approach allows a more comparable evaluation of the evolution of these two viruses during this critical phase of infection. The comparison between the viruses reveals differences in their mode of evolution, including in response to T-cell immune pressure.

## Results

### Mutations during acute and early HCV infection

In this analysis, mutations are defined as non-synonymous nucleotide changes that occur during infection relative to the first sampled time-point (T0). For many subjects this first time-point is within 3–4 months after estimated HCV exposure (median 10 weeks, mean 11.5 weeks) and often coincides with clinical acute hepatitis and jaundice. Subsequent follow-up sampling occurred at a median of 56 weeks (mean 61.2 weeks). All 21 study subjects were infected with genotype 1 and clinical parameters and sampling information for each subject is listed in Table 1. To increase subject numbers, four subjects from the Kuntzen et al study [13] were included in this analysis.

**Table 1:** Subject characteristics.

Subject ID	Sampling timepoints	GT	HLA-A	HLA-B	Outcome
	(weeks)#				
SR1	11	1a	1/1	8/44	SR
SR2	11	1b	2/25	15/44	
SR3	12	1b	3/24	15/35	
TR1	7, 20	1a	1/24	8/57	TR
TR2	13, 21, 25	1a	2/26	38/50	
TR3	8, 26, 32	1b	2/3	7/7	
TR4	19, 21, 44, 60	1b	2/24	18/44	
TR5	8, 14	1b	2/28	27/51	
TR6	9, 27	1b	3/25	7/18	
TR7	7, 8	1b	3/24	7/1501	
C1	14, 23, 38, 45, 92, 127, 193, 232	1a	2/24	18/44	C
C2*	15, 19	1a	26/26	49/55	
C3	12, 58, 78, 143	1a	11/31	40/51	
C4	9, 14, 76	1a	2/3	7/44	
C5	8, 12, 37, 56	1b	29/68	7/14	
C6	21, 82, 109, 121	1b	2/24	18/60	
C7	32, 63	1b	2/23	41/44	
BR554	8, 28, 60, 76	1a	2/31	39/51	
BR111*	10, 30, 58, 94, 118	1a	2/23	44/44	
03–32	8, 36, 106	1a	1/3	8/35	
BR1427*	0, 4, 22, 56	1b	26/29	15/15	

# Time since infection was estimated from the known time since onset of symptoms corresponding to approx. 7 weeks or seroconversion corresponding to 6 weeks.

*Acquisition of HCV via needle-stick injury or surgery. SR-Spontaneous Resolver, TR-Therapy Resolver, C-Chronic, GT-genotype. BR554, BR111, 03-32 and BR1427 are from [13].

Analysis of the HCV non-structural protein NS2-NS5B sequences from sequential time-points of subjects with acute and early HCV infection revealed a total of 170 amino acid changes, the majority of which were maintained into chronic infection (108/143 mutations in subjects with multiple time-points; Table S1). We then examined each of the proteins separately and found, consistent with other studies [22], [23], that NS2 exhibited the greatest variability within a subject, with NS3 being the most conserved protein (mean genetic distance for genotype 1a subjects NS2 = 0.019–0.06, NS3 = 0.005–0.018, NS4 = 0.006–0.026, NS5A = 0.02–0.045, NS5B = 0.01–0.019).

To determine if there was a higher rate of change during the acute phase compared to later time-points, as previously suggested [13], we separated the sampling time-points into three intervals 0–24 weeks, 24–48 weeks and 48–72 weeks from time of infection. For each individual we considered the cumulative genetic distances for both changes incurring amino acid change (nonsynonymous mutation) and silent (synonymous) mutations at each time-point (Table S2). Based on this analysis we found no evidence of differential rates of change in the three time intervals for either nonsynonymous (p = 0.18) or synonymous changes (p = 0.43). We did not have multiple time-points from individuals that naturally resolved infection and hence we are observing viral evolution in the context of a failed immune response with or without interferon alpha treatment.

### Mutations occur at sites likely to be under host HLA allele-specific immune pressure (viral adaptation)

To investigate the level of change in regions targeted by HLA-specific T-cell pressure, we counted the number of mutations within described T-cell epitopes (Los Alamos http://hcv.lanl.gov [18] and IEDB http://www.immuneepitope.org [19]) and putative T-cell targets marked by adaptation sites from genetic-based studies [20], [22].

Of the total number of mutations observed, 25.3% (43/170) occur at sites likely to be under HLA-specific CD8^+^ T-cell immune pressure (Table S1). Overall, the HCV proteins differ in the percentage of mutations presumed to be under T-cell immune pressure (12% for NS2, 46.4% for NS3, 21.4% for NS4, 24.4% for NS5A and 22.4% for NS5B) but this probably reflects the number of known and predicted T-cell targets in each protein examined in this study (17 for NS2, 47 for NS3, 25 for NS4, 19 for NS5A and 25 for NS5B). Most of these mutations are maintained into the later phase of infection. In addition, we observe mutations as late as 92 weeks after the onset of symptoms and just after the cessation of therapy (eg C1 NS5B) within published HLA-A and -B-restricted epitopes (Table S1), indicating ongoing immune pressure or compensatory mutations late in infection and during or after interferon treatment.

The proportion of mutations falling within known and predicted T-cell targets varies substantially between subjects from 0% to 100% (Table S1). Although the examination of viral adaptation sites as listed by recent genetic studies [20], [21], [22] allows for the identification of putative sites under immune pressure for HLA types not commonly represented in the literature as well as those epitopes that are more variable and hence may not be detected with more traditional cellular approaches, the number of known and predicted viral immune targets for each subject still differs depending on their HLA repertoire (Table S1). Subjects with less common HLA types will inevitably have less T-cell targets in our pre-defined list and tend to correspond to values at the lower end of this range.

### Existing HCV adaptation to the host's HLA-restricted immune response at the first sampling time-point

**Figure 1:**
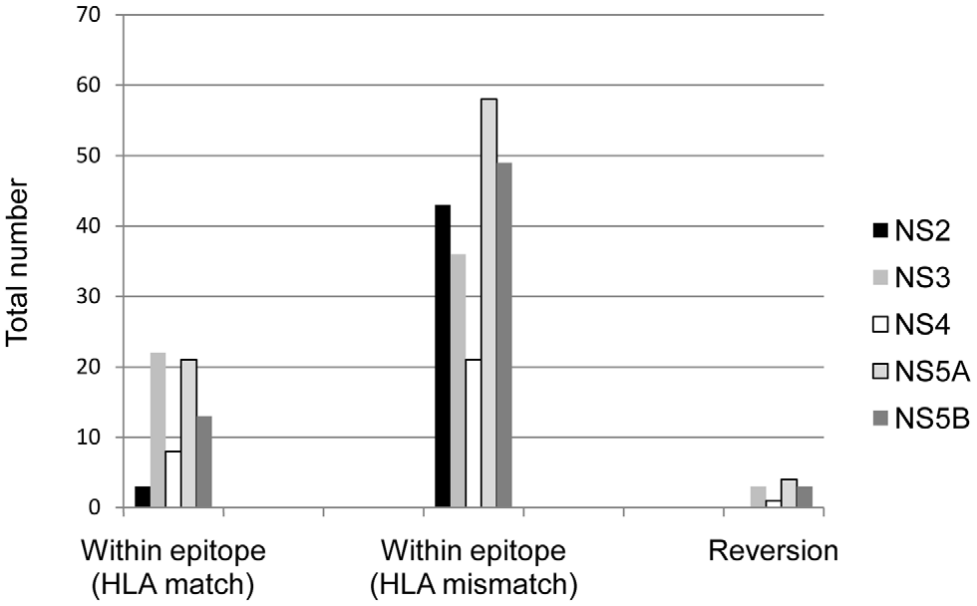
Existing sequence variations within CD8^+^ T-cell epitopes. Number of existing variations at first sampling time-point within CD8^+^ T-cell epitopes for matching HLA types and non-matching HLA types (relative to “wildtype” = HCV reference) and reversions in absence of specific HLA-restricted immune pressure.

The number of viral adaptations already present within the virus at the first sampling time-point is shown in Figure 1 and Table S3. For many subjects, the first sampling time-point was within 2–4 months of infection and existing viral adaptations could have resulted from mutations very early in the acute phase of infection. However, subject BR1427 already has an existing mutation within an HLA-matched epitope in NS2 close to the time of infection, which is unlikely to be due to T-cell pressure at this stage and may represent the transmission of a viral adaptation induced in the previous host. For viral adaptations within known T-cell epitopes and putative viral adaptation sites evident at the first sampling time-point, these amino acids are predominantly maintained throughout infection.

There is no obvious difference in the amount of viral adaptation at the first time-point between subjects that go on to become chronically infected and those who spontaneously clear the virus, although limited numbers here for each outcome restrict a more formal comparison.

### Amino acid reversion of viral adaptations in non-HLA matched subjects occurs rarely during the course of infection

We examined reversion to consensus (non-adapted) sequence of incoming variations within epitopes and at putative viral adaptation sites that were restricted by HLA alleles not present in the subject (Figure 1 and Table S3). In NS3 alone, a total of 35 variations within known T-cell epitopes or at putative viral adaptation sites restricted by HLA types that are not present in the newly infected hosts existed at the first time-point of which only 3 reverted to non-adapted sequence. Overall there was limited reversion observed in all subjects (11/207 revert to wildtype; Figure 1 and Table S3). If these reversions are added to the number of mutations associated with T-cell immune pressure it slightly increases the proportion from 25.3% to 31.8%.

The lack of reversion within HLA-restricted HCV T-cell epitopes and viral adaptation sites may suggest that mutations at these sites have limited impact on viral fitness or the cost is offset by compensatory mutations that prevent simple reversion. For some of these changes it may be that they do not revert over-time because they may not necessarily affect peptide presentation or T-cell receptor binding and do not incur a fitness cost as previously discussed.

### Preservation and accumulation of mutations in potential protective epitopes

The apparent lack of reversion described above for HCV may lead to the transmission of sequences harbouring mutations within epitopes that facilitate escape from protective CD8^+^ T-cell responses. In HIV infection the accumulation of mutations within epitopes has been previously described and it has been postulated that it may be required for ultimate viral escape [24], [25]. shows the HLA-B27 NS5B epitope which has previously been associated with protection [26] against HCV genotype 1. There are three sites within this epitope that are associated with HLA-B27 using a genetic approach [22] suggesting the need for multiple changes within the epitope to allow escape. Mutations at these sites within this T-cell epitope (compared to the known wildtype sequence) occur even in subjects that are not HLA-B27 positive and are maintained, facilitating an enrichment of variation. However, it is only in subject TR5 who expresses HLA-B27 that all three viral adaptation sites exist as the escaped form. A recent study by Dazert and colleagues has shown that the accumulation of mutations within the HLA-B27 NS5B epitope is required for escape from T cell recognition [27]. Furthermore, analysis of their own published data showed that variation versus consensus at three sites within this epitope is not observed in acute infected subjects who are HLA-B27 negative as also shown in our study.

**Figure 2:**
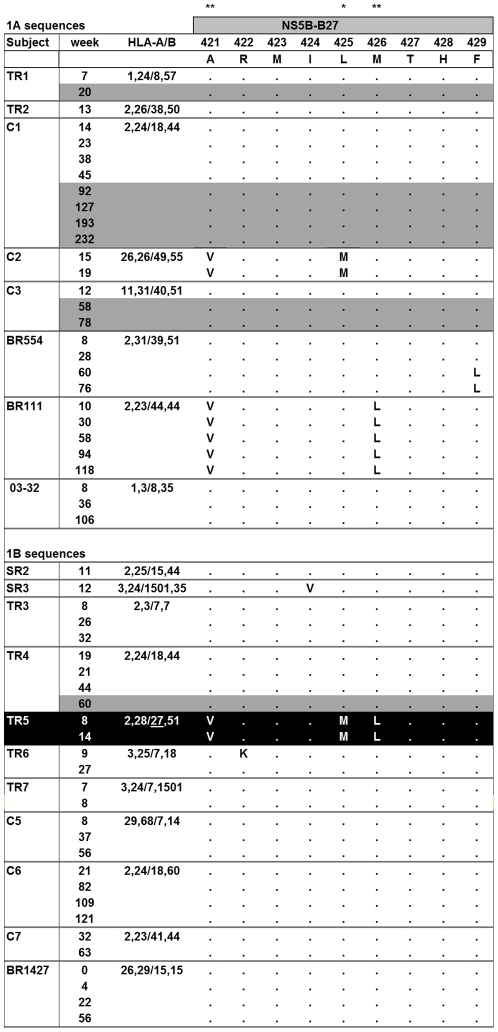
HCV sequences in different subjects for the HLA-B*27 epitope in NS5B. Single asterick indicates site of HLA-association based on 2-digit HLA typing and double asterick based on 4-digit HLA typing. Dots indicate identity to consensus. Numbering indicates position with the NS5B protein. Black indicates subject with HLA-B*27. Dark grey box indicates time-points after cessation of therapy.

### Pattern of viral evolution during acute to early chronic infection in HCV differs to that observed for a similar phase in HIV infection

**Figure 3:**
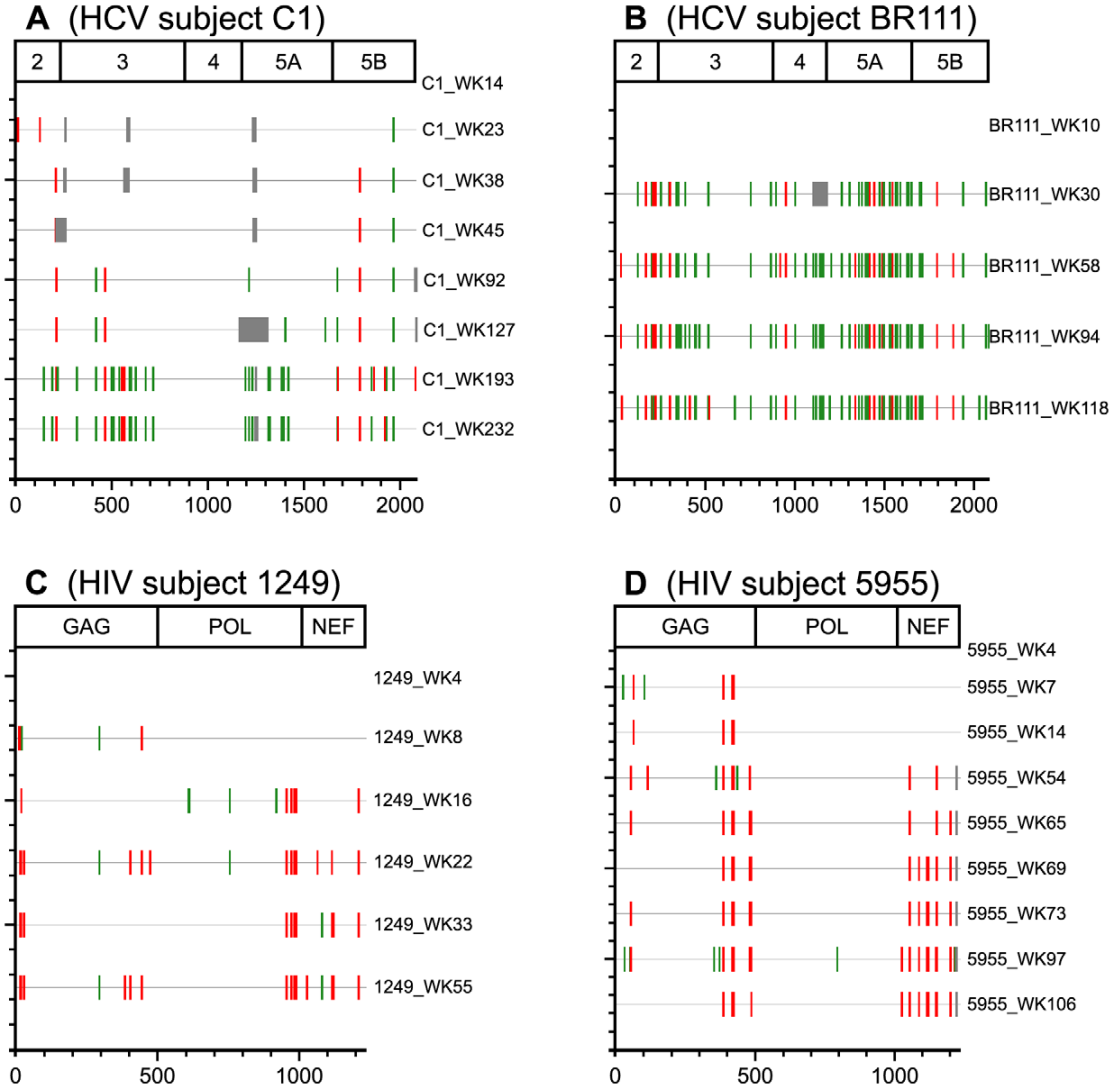
Plot of synonymous and non-synonymous changes during acute and early infection for HCV (A and B) and HIV (C and D). Representative subjects are shown that had similar sampling time-points. Time-points for HIV subjects were determined by adding 4 weeks to first time-points to represent time since infection.

The appearance of viral adaptations to a host's immune response likely reflects the onset of the immune response in the individual, the intrinsic mutation rate of the virus, the balance between benefit and fitness cost of mutation(s) and codon usage within selected infecting virus(es). Functional constraint (amount of intolerance towards nucleotide substitution) within the viral genome can be estimated by the rate of nonsynonymous and synonymous substitutions. In this approach, synonymous substitutions are thought to be neutral and representative of the mutation rate while the nonsynonymous rate reflects the balance between the mutation rate and purifying selection of deleterious mutations. [28]. We compared these changes in HCV and HIV, as the ability of the viruses to mutate to adapt to host immune pressure or revert to wildtype in the absence of specific immune pressure appears to differ and may reflect different constraints on these viruses. The HCV sequences described above were compared to the HIV sequences obtained from the recent study by Brumme and colleagues [14] during a similar phase of infection (median 425 days follow-up) and the results revealed different patterns of mutation as reflected in the number and rate of nonsynonymous and synonymous change (Figure 3 and Table 2). For HCV, rate and number of synonymous changes are greater than that observed for nonsynonymous changes. In contrast, HIV has a greater number (and in some cases the rate) of nonsynonymous changes than synonymous changes. The differences in the pattern of evolution for the two viruses is consistent with what is observed when you compare viral sequences from cross-sectional studies of chronic HIV [29] and HCV [22] subjects from the same geographical area and using similar sequencing methods (Table 2).

**Table 2:** Mean within and between individual synonymous and nonsynonymous rate for HIV and HCV*.

Virus	Protein	acute (n)	dN	dS	dS-dN	chronic (n)	dN	dS	dS-dN
HCV									
	NS2	8	0.0019	0.0038	0.0021	49	0.041	0.194	0.153
	NS3	9	0.0011	0.0051	0.004	50	0.011	0.2	0.189
	NS4	9	0.0015	0.01	0.0085	39	0.014	0.213	0.198
	NS5A	14	0.0018	0.0092	0.0074	48	0.03	0.178	0.149
	NS5B	11	0.0016	0.0072	0.0056	41	0.014	0.135	0.121
HIV∧									
	GAG	96	0.0018	0.0019	0.0001	138	0.041	0.126	0.085
	POL	97	0.001	0.0019	0.0009	130	0.031	0.112	0.08
	NEF	92	0.0062	0.0042	−0.002	105	0.103	0.151	0.048

*dN and dS computed using the Modified Nei and Gojobori method (MEGA v4). Intra-individual values for acute infection cohorts and inter-individual values for chronic infection cohorts. Two time-points used for analysis - 1st time-point (T0) within 3–4 months of infection and 2nd time-point closest time-point to 1 year from T0. n = number of subjects.

∧ HIV sequences from [14]. dS-dN summed for all subjects and then divided by number of total subjects (for acute subjects). HCV genotype 1a sequences from cross-sectional chronic cohort in [22]. HIV clade B sequences from cross-sectional chronic cohort in [29].

The number of putative viral adaptations found in this study is consistent with others [9], [13] but is less than that observed for HIV during a similar phase of infection (30–60% for Gag, Pol and Nef) [14]. In the present study we defined adaptation as change within a published T-cell epitope (n = 96) or at a putative viral adaptation site (n = 36) in the NS2-NS5B region of which 9 sites were within known HCV T-cell epitopes. In the recent Brumme et al study on HIV [14] the authors defined adaptation as change occurring at an adaptation site within a known T-cell epitope (n = 71). The adaptation sites in the HIV study were identified using the same approach as described for HCV. Although the present study attempts to allow a comparable analysis of the evolution of the two viruses by using the same analytical approaches, some of the mutations within known HCV T-cell epitopes may not necessarily be associated with specific host immune pressure (as discussed earlier). However, irrespective of this limitation, it does appear that the proportion of mutations associated with T-cell pressure is higher in HIV (30–60%) than for HCV (31.8%).

## Discussion

Acute HCV infection is difficult to study due to the lack of clinical disease around the time of viral inoculation. This study aims to assess viral evolution during the first months of infection and at a time when clinical disease of hepatitis and/or jaundice is present. Here, we find evidence of HCV adaptation during the acute phase of infection, maintenance of these changes into chronic infection and little evidence of early reversion.

Overall, HCV evolution occurs to a limited extent and accumulates overtime. Part of these results may have been expected as HCV infection is associated with a narrow and often functionally impaired T cell response that can be maintained, albeit at a low level and detectable mainly in hepatic lymphocytes during persistent infection [30], [31], [32]. The maintenance of viral adaptations may therefore reflect ongoing T-cell pressure in chronic HCV-infected subjects. Alternatively, preservation of changes could be the result of linked compensatory mutations that stabilize the primary escape mutation and/or improve viral function. On the other hand, these mutations may be well tolerated by the virus and have no or little impact on viral fitness and function. Functional studies assessing viral replication may clarify further the importance of these mutations.

Surprisingly we were unable to show a clear difference between the rate of viral evolution during acute and the chronic phase of infection, as generally the early phase of infection is associated with a stronger T-cell response that can coincide with clinical hepatitis and jaundice [5], [30]. However, adaptations were already present in several subjects at the first sampling timepoint and some of these may have occurred before the first sampling timepoint.

We also found examples where mutations within known T-cell epitopes occur at later time-points during early chronic phase and particularly in one case after cessation of treatment. These late changes may reflect new emerging T-cell pressure targeting novel viral regions at later stages of infection or the ongoing “editing” of the HCV genome with secondary mutations stabilizing primary escape mutations, potentially increasing viral fitness. Interestingly, previous studies on HCV have found little or no evidence of new T-cell responses after the first six months of infection [13], [30]. Furthermore, the appearance of changes after treatment cessation in one case suggested that treatment with IFN-alpha may shift the distribution of viral populations via the reduction of viral heterogeneity through bottlenecks and/or increased T-cell pressure on the virus during therapy. Further studies are needed to clarify the effect of therapy on viral adaptation.

The lack of reversion over-time of pre-existing viral adaptations in subjects without the relevant HLA type suggest that these changes may require compensatory mutations that do not allow simple reversion, therefore allowing the accumulation of mutations and transmission of pre-adapted viruses. In contrast, a large number of the mutations associated with CD8^+^ T-cell pressure observed during the acute phase of HIV infection in the Brumme et al study were reversions in the absence of HLA-restricted immune pressure (∼70%); many of which are escape mutations known to incur a fitness cost [14].

In this study, we were unable to directly assess if the changes observed in subjects were within regions under CD8^+^ T-cell pressure using cellular assays due to the lack of available peripheral blood cells. Accordingly, changes in described epitopes and adaptation sites included in this analysis are assumed to represent viral escape mutations in the study subjects. We acknowledge that some of these changes may not be true escape mutations, however, many of the predicted adaptations from the genetic study fall at known escape sites [20], [22] and Figure S1 shows a diminished IFN-gamma response to a variant peptide with a novel variation (observed in subjects C1 and C4) in a described epitope compared to the consensus peptide in samples from a chronically HCV infected subject (not part of the acute study cohort). Furthermore, previous studies examining mutations associated with detectable CD8^+^ T-cell responses during acute HCV infection showed that a number of variant peptides (most unconfirmed escape mutations) of described epitopes resulted in either loss or reduction of T-cell recognition [9]. Taken together, these results suggest that many of the variants observed within known epitopes and at adaptation sites in this study are likely to impact on T cell recognition.

Despite limitations in the study, the 25.3% adaptation rate obtained in this study is comparable to the rate obtained by Kuntzen et al (11% overall and 20.8% during the acute phase for non-envelope mutations; [13]) on four subjects during acute HCV infection that were also analysed in this study. Kuntzen and colleagues were able to directly correlate CD8^+^ T-cell responses to peptides covering the HCV genome with sequence evolution in the autologous virus and, for the most part, reduced T-cell recognition for the variant peptide. In addition, an earlier study by Cox and colleagues on HCV evolution during the first six months after acute infection showed a comparable rate of 25% of mutations were in detected CD8^+^ T-cell responses [9], although this number was about 50% when only mutations outside the envelope were included.

Finally, when the rate and number of amino acid changes relative to silent mutations is compared between HIV and HCV in a similar phase of infection, HCV appears to have a greater proportion of silent mutations than HIV. In addition, the observation of a higher proportion of mutations associated with CD8^+^ T-cell pressure, including reversions, in HIV compared to HCV highlights key differences in the strength of selection pressures on these two viruses, that is their plasticity and ultimately their ability to escape CD8^+^ T-cell responses. Understanding viral escape dynamics in early infection in these two infections is of primary importance for effective T cell vaccine design and may also imply different drug resistance dynamics in HIV and HCV when exposed to anti-viral drugs.

## Materials and Methods

### Subjects

Plasma samples from 17 individuals with acute HCV genotype 1 infection (7, 1a; 10, 1b) were collected at the Klinikum Grosshadern in Munich, Germany between 1994 and 2005. Acute or early phase of HCV infection was defined as 4 months from the onset of symptoms, commonly jaundice or as 6 months after a known infection event. After acute infection, three individuals spontaneously resolved their HCV infection (SR), 7 cleared after IFN-α based therapy (TR) and 7 progressed to chronic infection. Table 1 shows details of the study participants. Two-digit HLA-A and -B typing and clinical data such as viral load (VL) and therapy details were available for each individual. In addition, sequences from four HCV genotype 1-infected individuals previously described by Kuntzen et al [13] were obtained from Genbank and were analysed with the samples obtained from the Munich subjects.

### Ethics Statement

All patients gave written informed consent to participate in the study and the protocol and the procedures of the study were conducted in conformity with the ethical guidelines of the Declaration of Helsinki and according to study protocols approved by the institutional review board (University Hospital Munich).

### Bulk HCV sequencing

Viral RNA was extracted from plasma samples using the COBAS AMPLICOR HCV Specimen Preparation Kit v2.0 (Roche) according to manufacturer's instructions. Bulk sequencing of HCV NS2-NS5 was performed as previously published [22]. Sequence coverage for each protein in each subject is shown in Table S4 (GenBank accession numbers HM746802-HM746915). Figure S2 shows the HCV NS3 and NS5B phylogenetic trees with the well-supported individual subject clusters and separation into genotype 1A and 1B groups. The trees also show the limited intra-subject diversity compared to the diversity observed between the subjects.

### HCV sequence analysis

#### Analysis of sequence evolution during acute and early HCV infection

For each individual we considered the cumulative genetic distances at each time point observed. The times were then broken into 0–24 weeks, 24–48 weeks and 48–72 weeks. Only a small number of cases had data beyond this so we only considered mutational change over 0–72 weeks and analysed the relative rates of increase in the three time periods. Estimates in the 0–24 week range were based on the period from first measure to 24 weeks and were scaled accordingly. It was assumed that the cumulative changes could be approximated by a continuous piecewise linear function with possible rate changes at 24 and 48 weeks. The analysis was carried out using linear mixed models incorporating correlations between sequence changes within the same protein and proteins within the same individual. Analyses were carried out in S+ and rates compared using likelihood ratio tests.

#### Genetic diversity

Intra- and inter-subject genetic distances were calculated using the modified Nei-Gojobori method for both synonymous and nonsynonymous sites using MEGA v4 software.

#### Highlighter plots

Aligned longitudinal sequences for both HCV and HIV-infected subjects were examined for synonymous and nonsynonymous changes using the highlighter program available at http:www/hiv.lanl.gov.

#### Phylogenetic analysis

Phylogenetic trees were constructed using the neighbor-joining method based on the Kimura-2-parameter model with 500 bootstrap replications. All analyses were performed using MEGA v4 software.

### Assessment of HCV adaptation

HCV sequences were aligned against the consensus sequences for genotype 1a and 1b obtained from previous genetic studies [20], [22]. Initially, the HCV genotype sequences were scanned for sequence changes within known HLA-A and -B-restricted epitopes within NS2-NS5 (Los Alamos and IEDB databases). Next, sequence changes were compared to four and two-digit HLA-A and -B-associated viral polymorphisms observed within HCV chronic infection cohorts [21], [22]. De-novo mutations were defined as newly occurring changes (as compared to the first sequence time-point) within known epitopes or at HLA-association sites relevant for the host's HLA. Reversion events were defined as changes back to the sequence at the initial time-point, which included at sites within or outside known epitopes or at HLA-association sites relevant for the host's HLA.

## Supporting Information

Figure S1 **Reduced IFN-gamma production for variant peptides of a known HLA-A2 epitope.** A. Viral sequence for two HLA-A2 subjects (C1 and C4). C1 had therapy from weeks 50-84. B. IFN-gamma responses to consensus and variant peptides in a chronically HCV infected subject with HLA-A2.
(XLSX)

Figure S2 **Phylogenetic analysis of NS3 (A) and NS5B (B) HCV sequences from individuals during acute and chronic infection.** Tree constructed using the neighbor-joining method based on the Kimura-2-parameter model with 500 bootstrap replications. All analyses performed using MEGA v4 software.
(PPT)

Table S1 **Viral evolution in acute and early HCV infection.**
(XLS)

Table S2 Synonymous and nonsynonymous genetic distance over time for NS2-NS5B*.
(XLS)

Table S3 **Pre-existing viral adaptation at first time-point and later reversion to wildtype.**
(XLS)

Table S4 **Sequence coverage for all subject time-points.**
(XLS)
