# Supplementary material for: Constrained Pattern of Viral Evolution in Acute and Early HCV Infection Limits Viral Plasticity
Source: PLoS One. 2011 Feb 8;6(2):e16797. doi: 10.1371/journal.pone.0016797 (PMC3035653; doi:10.1371/journal.pone.0016797)
Supplement: Figure S2 — Phylogenetic analysis of NS3 (A) and NS5B (B) HCV sequences from individuals during acute and chronic infection. Tree constructed using the neighbor-joining method based on the Kimura-2-parameter model with 500 bootstrap replications. All analyses performed using MEGA v4 software. (PPT) [file pone.0016797.s002.ppt]

## Slide 1
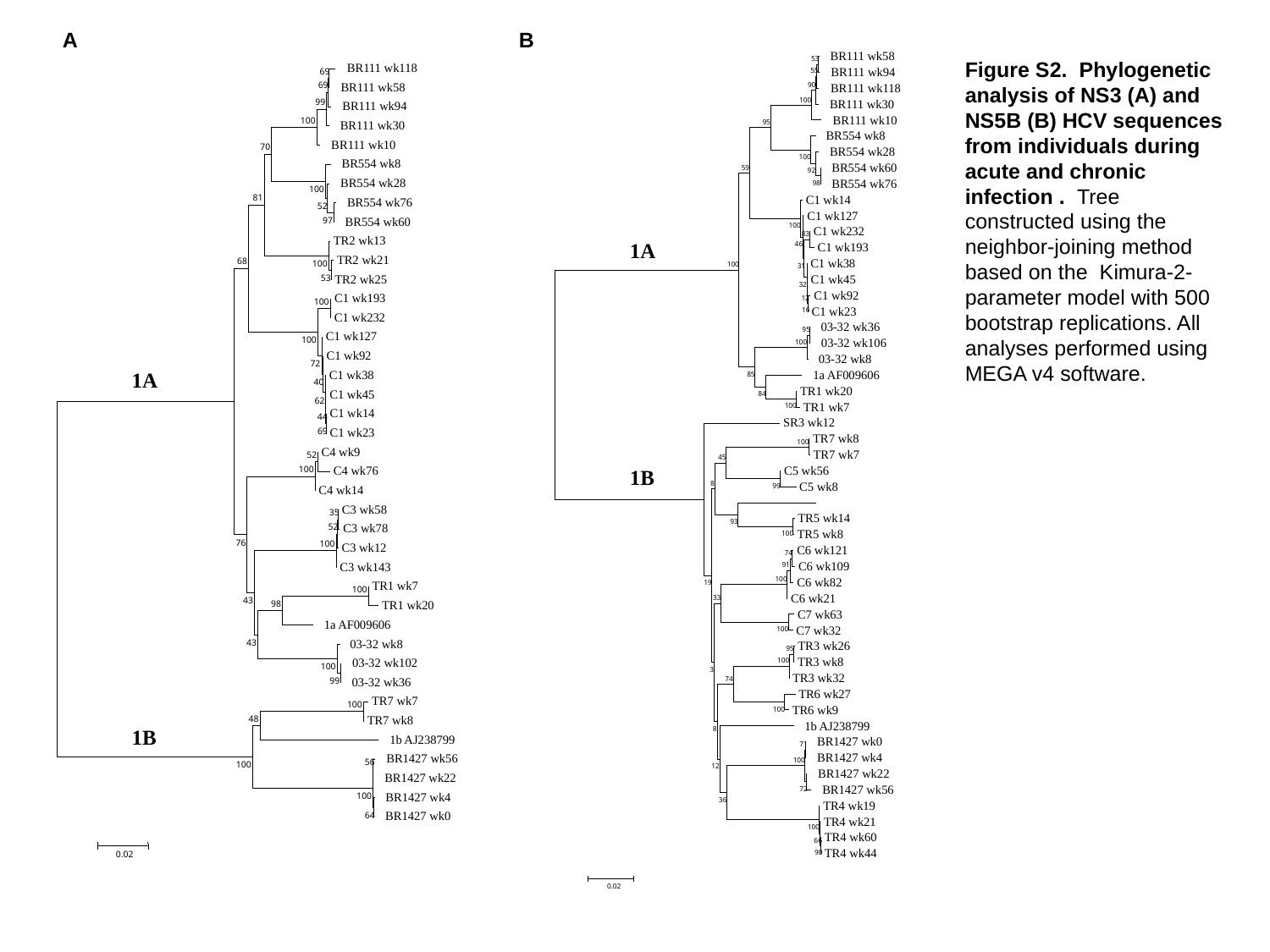

A
B
0.02
 BR111 wk118
69
 BR111 wk58
69
99
 BR111 wk94
100
 BR111 wk30
 BR111 wk10
70
 BR554 wk8
 BR554 wk28
100
81
 BR554 wk76
52
 BR554 wk60
97
 TR2 wk13
 TR2 wk21
68
100
 TR2 wk25
53
 C1 wk193
100
 C1 wk232
 C1 wk127
100
 C1 wk92
72
 C1 wk38
40
 C1 wk45
62
 C1 wk14
44
 C1 wk23
69
 C4 wk9
52
 C4 wk76
100
 C4 wk14
 C3 wk58
35
 C3 wk78
52
76
100
 C3 wk12
 C3 wk143
 TR1 wk7
100
43
 TR1 wk20
98
 1a AF009606
 03-32 wk8
43
 03-32 wk102
100
 03-32 wk36
99
 TR7 wk7
100
 TR7 wk8
48
 1b AJ238799
 BR1427 wk56
56
100
 BR1427 wk22
 BR1427 wk4
100
 BR1427 wk0
64
0.02
 BR111 wk58
53
 BR111 wk94
55
 BR111 wk118
90
100
 BR111 wk30
 BR111 wk10
95
 BR554 wk8
 BR554 wk28
100
 BR554 wk60
59
92
 BR554 wk76
98
 C1 wk14
 C1 wk127
100
 C1 wk232
83
 C1 wk193
46
 C1 wk38
100
31
 C1 wk45
32
 C1 wk92
12
 C1 wk23
16
 03-32 wk36
95
 03-32 wk106
100
 03-32 wk8
 1a AF009606
85
 TR1 wk20
84
 TR1 wk7
100
 SR3 wk12
 TR7 wk8
100
 TR7 wk7
45
 C5 wk56
 C5 wk8
8
99
 TR5 wk14
93
 TR5 wk8
100
 C6 wk121
74
 C6 wk109
91
 C6 wk82
100
19
 C6 wk21
33
 C7 wk63
 C7 wk32
100
 TR3 wk26
95
 TR3 wk8
100
3
 TR3 wk32
74
 TR6 wk27
 TR6 wk9
100
 1b AJ238799
8
 BR1427 wk0
71
 BR1427 wk4
100
12
 BR1427 wk22
 BR1427 wk56
72
36
 TR4 wk19
 TR4 wk21
100
 TR4 wk60
66
 TR4 wk44
90
Figure S2. Phylogenetic analysis of NS3 (A) and NS5B (B) HCV sequences from individuals during acute and chronic infection . Tree constructed using the neighbor-joining method based on the Kimura-2-parameter model with 500 bootstrap replications. All analyses performed using MEGA v4 software.
1A
1A
1B
1B
